# Which Shall It Be, M.D. or A.B.?

**Published:** 1902-07

**Authors:** Harry L. Grant

**Affiliations:** Providence, R. I.


### WHICH SHALL IT BE, M.D. OR A.B.?

BY HARRY L. GRANT, D.M.D., PROVIDENCE, R. I.

Every man holds up to himself an ideal towards which he works. That ideal may be relatively high or low, depending on the individual moral standard ambition and desires, yet it exists, whether he recognizes it or not. Hence to have any sort of a definition as to what constitutes the "ideal dentist" is out of the question, as there would be about as many definitions as there are individuals.

Considering the difference of opinion as to the final product, it is not strange that opinions differ so widely as to what constitutes the best means to attain the end, the best training for the student to pursue to develop in himself the necessary qualities required of the successful dental practitioner of to-day.

It may be said that nearly all agree upon one point,—namely, that the dental student should receive some general preliminary education before entering upon this specialty. Among a few that preliminary education is made to include a degree. But here the ways diverge, some claiming that to attain the best results the degree should be such a course as is represented by M.D., and others that it should be such a course as is represented by A.B. or its equivalent.

It is difficult to pass judgment without being biassed by one's opportunities or lack of opportunities in life, and it is probable that no two individuals require exactly the same educational training. Those who have been fortunate enough to have the medical and then the dental training look at dentistry from a physician's point of view. It being a branch of medicine to them, places the dentist on the same plane as the oculist, aurist, gynæcologist, and so on, and one would hardly think of taking up those branches without first having a full medical course. Had dentistry grown up under the fostering care of the medical school, undoubtedly it would be approached through the same channel as the others, but inasmuch as it has grown by its own efforts, and has assimilated much from the medical, it does seem as if dentistry could not be quite classified beside the other specialties. So marked is the mechanical side that by many in times past it has been regarded as a trade. On the other hand, as the importance of its relation to medicine has grown, it has been regarded by many as purely a department of medicine. While the other specialities require just as skilful manipulation, there is not that daily and constant working with instruments that is necessary in dentistry, under all kinds of conditions, and that, too, on the very hardest tissues of the body. There is not that daily work in the laboratory the ability to perform which is supposed to be possessed by every dentist. We have, in marked contrast, the trade and the profession, the expert mechanical worker and the intellectual diagnostician.

If one is to be an oral surgeon, a stomatologist in the full meaning of the word, a more general medical training is undoubtedly beneficial, and even then would it not be better if the medical came after the dental training? Why? Because the average medical practitioner looks upon dentistry as a specialty which can be taken up readily without much daily manipulation of instruments, so long as one knows what ought to be done; whereas, the one who takes up dentistry as such first devotes many hours daily to the training of one's fingers. In other words, the physician is apt to exaggerate the medical without due attention to the mechanical, whereas the student is more apt to bestow his special attention to the mechanical side when his manipulative skill is most plastic.

For every gain there is a loss, for every loss a gain; whatever course a student takes he gains something he would not had he taken other, and also loses something he would not had he pursued some other. The student who takes a medical course after having had the slight preliminary training required to enter many medical schools should gain much in time and medical knowledge over him who takes an A.B. or its equivalent. Indeed, whatever course a man pursues he uses to some extent all the knowledge he ever acquires, however remote it may seem from the work in hand. The M.D. in dentistry applies his medical knowledge and undoubtedly gains much thereby.

The A.B. in dentistry applies his previous training in many ways. So apparent are the many benefits, that it is hardly necessary to mention those which accrue to the dental student who has had a medical training. He is a physician. He can diagnose any of the better-known diseases. He is proficient in auscultation and percussion. He feels perhaps as competent to perform an appendix operation as to extract a tooth. He has a foundation of medical knowledge which the average dentist has not. But a great deal of his medical acquirements will not be in daily use, and, like all knowledge which is not in constant use, the unused medical training will soon find its way to the top shelves of the mind, there to become covered with dust and soon a back number.

Knowledge is not all, medical knowledge is not all, that is needed to raise the dental profession as it is to-day to that ideal position in which we would like to place it to-morrow. Of what, then, is the profession in great need? Of cultured, broad-minded, studious men, who have the foundation and the will to work unselfishly for the advancement of the profession from the date of their entrance into it until their death. There have been and there are many such men in the dental profession. There is room for, and there is need of, many more such men.

The type of man who goes to college differs in a very general way from the type of man who omits the college course and goes directly into a profession or business. The physician who has served as interne in one of our best hospitals differs from the physician who steps directly from the medical school to a private practice.

Reason is an attribute of experience, as energy is of matter. The man of broad experience has greater resources upon which to draw in life's work than the same man could have with a very limited experience. Within the college course is crowded a world of objective impressions at a time when the mind is most easily impressed. These serve as a very firm foundation upon which the individual builds his life-work. There will always be professional schools which do not require much preliminary education. Why not make a special effort to have a few in dentistry which will especially attract the college man?

The professional school is primarily for the trained mind, for the mind which is endowed with or has acquired the power of concentration, of continuous thoughtful application, of adaptation, of assimilation, and for the mind which has many other attributes which in the average man are developed in a regular college course. President Faunce, of Brown, has said, "There are two objects in education,—one is the training for power, the second is training for vocation. The first has been followed in colleges for generations back. I myself tell my students to follow useless studies for useful purposes. They are summed up as giving power without regard to what the boy or girl is to do in the world."

Books alone will never train fingers and develop mechanical skill. It is the doing of the thing with thoughtful intelligence back of it that brings about manual dexterity. Inclination and some intuitive mechanical ability are essential, and the mind with these attributes will become a more useful member in society if the education previous to taking up the profession has been broad and *not necessarily purely practical.*

The methods of instruction in the professional school and those in college are so entirely and essentially different that one can readily see why the average individual does not get the same mental training in the one as in the other. The college is the link in the chain which connects the boy with the man. For four years he is associated intimately with those who are to follow various pursuits. Some of his associates excel in the various departments,—some in mathematics, some in languages, some in history, some in literature, some in mechanics, and so on. By these very associations his mind is broadened so that he has a better view of life; by them he is stimulated to develop the different departments of mind just as the athlete develops the different muscles of the body. It is the daily application of the mind which brings about the difference between the real student and the mind-wanderer. Probably two-thirds of those who enter college have no more than a vague idea as to their life-work. A few have marked tendencies in some one direction. Others are groping to find in themselves that for which they are best fitted. Before long a kind of intuition will direct the one who is in earnest in studying his own fitness and desires. When that is once settled he elects readily such subjects as have a bearing upon his life-work, not, however, to the exclusion of other subjects which help to make a well-rounded mind. He does not become one-sided, as he might in a purely professional course. The student who so elects in a well-equipped college can have four years of comparative anatomy and physiology, in which is included dissecting and other manipulative work, several courses in chemistry, physics, botany, histology, geology, and so on, but not to the exclusion of mathematics, languages, philosophy, English, history, and literature. And furthermore he will have a daily recitation and training of mind such as he cannot have in a professional school, simply because the professional school is a special means to a special end, and cannot devote the time directly to the training for mental power.

There is no attempt intended to claim that all college men have better-trained minds than all non-college men. All educated men *do not come from college.* They may not need the training as they may acquire it in other ways. However, many educated men do come from college, and do occupy positions which they could not without having had such opportunities. It may be years before the real benefits of college training are manifest, but sooner or later the one who has had that privilege will feel that he has a solid foundation upon which to build the superstructure, his special work in life.

Culture, the taking of the raw material and developing it to its fullest capacity, and refinement, the separating out of the undesirable qualities and reducing the desirable to the finest possible texture, as applied to the mind are difficult terms to define, and yet every man realizes and feels the difference between the possession of them and the total lack of them. When the average dentist is cultured and refined in the sense which we all know and feel, there need be no fear that he will not be recognized to be on an equal footing as a professional man with his brother physician, lawyer, clergyman, or college professor. When the average dentist is cultured and refined, there will be less tendency towards a spirit of commercialism, a spirit of petty jealousy, a want of high ethical and moral standing.

There is a thought expressed in a number of *Success* which has a bearing on this subject, and if we may we will quote it here: "We neglect, at our peril, the brain or the muscles alike. A monstrosity is not a man. To cut off the physical, the moral, or the spiritual branches of the tree of life, or to let them die by withholding their natural nourishment, or to allow one's being to develop a large, one-sided brain-gland by pursuing some mental specialty at the expense of everything else, may produce one who will stand high as a specialist, but will never produce *a man.*"

Are not we as a profession in greater need of the *man* well developed in every particular, rather than of the dentist more highly specialized? By which course can it be best attained?

